# The outpatient economic burden of respiratory syncytial virus infection in adults aged ≥ 60 years in Germany: results from the BUCOSS study

**DOI:** 10.1186/s12931-026-03866-1

**Published:** 2026-08-14

**Authors:** Roland Diel, Paul Schmidt, Christopher Alexander Hinze, Grit Barten-Neiner, Frank Eberhardt, Björn Martens, Christoph Wyen, Tobias Knaupp, Rachel Reeves, Alen Marijam, Gernot Rohde

**Affiliations:** 1 Institute for Epidemiology, University Hospital Schleswig-Holstein, Campus Kiel, Germany; 2 Statistical Consulting for Science and Research, Berlin, Germany; 3 Department of Respiratory Medicine and Infectious Diseases, Hannover Medical School, Hannover, Germany; 4 CAPNETZ STIFTUNG, Hannover, Germany; 5 Lungenpraxis Hohenzollerndamm, Berlin, Germany; 6 Praxis am Ebertplatz, Cologne, Germany; 7 Department I of Internal Medicine, Division of Infectious Diseases, University Hospital of Cologne, Cologne, Germany; 8 MVZ Dr. Renard und Kollegen, Nürnberg, Germany; 9 GSK, Philadelphia, USA; 10 GSK, Munich, Germany; 11 Department of Respiratory, Faculty of Medicine, Intensive Care and Sleep Medicine, Marburg-University, Marburg, Germany

**Keywords:** Respiratory syncytial virus, Older adults, Outpatient care, Cost of illness, Societal costs, Productivity loss, Acute respiratory infection, Vaccination

## Abstract

**Background:**

Respiratory syncytial virus (RSV) is a major cause of acute respiratory infection (ARI) in adults aged ≥ 60 years, but data on its outpatient epidemiology and societal costs remain limited.

**Methods:**

BUCOSS was a prospective observational study conducted over 3 seasons (2022/23–2024/25) in adults aged ≥ 60 years presenting with ARI to 29 outpatient practices in Germany. Multiplex PCR testing was performed at baseline. RSV-positive patients were prospectively followed for up to 28 days. Clinical and socioeconomic burden, including direct medical, indirect, and patient-borne out-of-pocket costs, was assessed using structured questionnaires on days 0, 14, and 28. Approximate national burden was estimated using incidence-based and ARE–prevalence-based extrapolation, including an additional scenario analysis excluding indirect costs.

**Results:**

Among 3,261 patients with ARI, 192 (5.9%) had PCR-confirmed RSV infection. RSV prevalence was 5.49%, and estimated RSV incidence was 1,030.6 cases per 100,000 population. After exclusion of 4 patients hospitalized at baseline, 188 patients were included in the outpatient cost analysis. Mean age was 71.6 (SD 8.52) years, 57.4% were female, and 60.1% met the study high-risk criteria. Mean societal cost per episode was €510.0 (SD €742.58); costs were highest in patients aged 60–64 years. Total costs comprised direct medical costs of €161.3 (SD €59.57), indirect costs of €331.8 (SD €746.75), and out-of-pocket costs of €16.9 (SD €22.25), corresponding to 31.6%, 65.1%, and 3.3% of total societal costs, respectively. Indirect costs were driven almost entirely by productivity losses. Approximately 40% of cases occurred in adults without study-defined high-risk status. The two extrapolation approaches yielded approximate estimates of 258,000–374,000 medically attended outpatient RSV cases per season among adults aged ≥ 60 years in Germany, corresponding to €131.7–190.9 million in societal costs. In a scenario analysis excluding indirect costs, the estimated burden decreased to €46.0–66.7 million per season.

**Conclusions:**

RSV is an economically important cause of outpatient ARI in older adults. These findings highlight a substantial outpatient burden beyond inpatient care and support RSV prevention strategies in aging populations.

**Supplementary Information:**

The online version contains supplementary material available at https://doi.org/10.1186/s12931-026-03866-1.

## Introduction

Respiratory syncytial virus (RSV) is increasingly recognized as an important cause of acute respiratory infection (ARI) in older adults. Although long regarded primarily as a pediatric pathogen, RSV can also cause substantial morbidity in adults aged ≥ 60 years, particularly in those with chronic cardiopulmonary disease [1, 2]. Clinical manifestations range from upper respiratory tract symptoms to lower respiratory tract disease, including pneumonia and exacerbations of chronic obstructive pulmonary disease (COPD) or asthma [3].

Recent epidemiological studies and systematic reviews indicate that RSV contributes substantially to respiratory morbidity and healthcare burden in older populations, including in Germany [4, 5]. In particular, Scholz et al. [5] showed, using German public surveillance and registry data, that adults aged ≥ 60 years accounted for approximately 13.0% of RSV-related outpatient consultations and, in the 2022/2023 season, for 12,800 RSV-related hospitalizations and 1,340 in-hospital deaths, corresponding to 24% of all RSV-related hospitalizations and 93% of all RSV-related in-hospital deaths. Despite this growing recognition, the burden of RSV in older adults remains incompletely characterized in the ambulatory setting. Routine virological testing is uncommon in outpatient care, and RSV symptoms are often non-specific and difficult to distinguish from those of other respiratory pathogens. Consequently, ambulatory RSV episodes are likely to be underdiagnosed and underreported [4–6].

Prospective data on the socioeconomic burden of ambulatory RSV infection in older adults are scarce. Available evidence is dominated by claims-based or hospital-based studies [7, 8], which mainly rely on administrative billing and insurance data and therefore provide only limited insight into the broader societal burden of disease.

This evidence gap is particularly relevant in Germany. Older adults are not only clinically vulnerable to RSV, but also remain economically active to a considerable extent. Germany has one of the highest employment rates among people aged 55–64 years in the European Union [9], indicating that a substantial proportion of adults approaching or exceeding 60 years of age remains part of the workforce. In this population, ambulatory RSV episodes may generate not only indirect costs through work absenteeism, but also patient-borne out-of-pocket expenses and time losses associated with support from relatives or accompanying persons during healthcare utilization. A meaningful assessment of the economic burden of ambulatory RSV in older adults should therefore not be restricted to routine administrative healthcare data alone.

A further challenge is the marked heterogeneity of published estimates of ambulatory RSV frequency in older adults across countries, study settings, and seasons. Reported incidence and prevalence estimates vary widely. For example, McClure et al. [10], in a community cohort from Wisconsin, reported an RSV incidence of 147 per 10,000 persons (95% CI 110–196) among adults aged 60–69 years, whereas Korsten et al. [11], in a prospective European cohort of community-dwelling older adults, reported seasonal cumulative incidences of RSV illness of 4.2% and 7.2% in two consecutive seasons.

Reliable country-specific estimates are therefore needed to translate per-case clinical and economic outcomes into population-level burden estimates and to inform the development and evaluation of RSV-specific preventive strategies, including vaccination.

To address these clinical and economic evidence gaps, we used data from the prospective multicenter BUCOSS study to assess the burden of PCR-confirmed RSV infection in adults aged ≥ 60 years treated in primary care and pulmonology practices in Germany between 2022 and 2025. Based on detailed prospective individual-level data collection, including a broad range of direct and indirect cost components, the study aimed to provide a comprehensive assessment of the societal economic burden of ambulatory RSV infection in older adults.

## Methods

### Study design and setting

The BUCOSS RSV 60 + study (“BUrden of Disease and COst of illneSS for RSV and other pathogens in patients 60 years and older within the outpatient setting”) is a prospective, multicenter, non-interventional observational study conducted in Germany to assess the clinical and economic burden of respiratory syncytial virus (RSV) and other respiratory pathogens among older adults treated in outpatient care. The study was prospectively registered in the German Clinical Trials Register (DRKS00030320) on 17 October 2022.

The study covered three consecutive respiratory infection seasons (2022/23, 2023/24, and 2024/25), each running from 1 October to 30 April, and included patients from 29 outpatient primary care and specialist practices across eight federal states in Germany (Baden-Württemberg, Bavaria, Berlin, Brandenburg, Hesse, Lower Saxony, North Rhine-Westphalia and Saxony-Anhalt), although not all sites participated in every season. Study sites were not selected using a population-weighted random sampling strategy. Instead, site selection aimed to capture routine outpatient care across geographically diverse regions and practice settings, including general practitioner and pulmonary specialist practices in metropolitan and rural areas.

### Study procedures and follow-up

Patients aged ≥ 60 years presenting with acute respiratory infection (ARI) in participating practices were enrolled after written informed consent. At baseline (Day 0), demographic and clinical data were recorded, including medical history, comorbidities, employment status, and health-related quality of life. A nasopharyngeal swab was obtained for multiplex RT-PCR testing for RSV A/B and a broader respiratory pathogen panel comprising SARS-CoV-2, human bocavirus, seasonal coronaviruses (229E, HKU1, NL63 and OC43), influenza viruses (A, A/H1N1, A/H3N2 and B), human metapneumovirus, parainfluenza viruses 1–4, human rhino-/enterovirus, Bordetella pertussis, Chlamydia pneumoniae, Legionella pneumophila and Mycoplasma pneumoniae.

High-risk status was defined as the presence of at least one of the following conditions: COPD, asthma, any chronic respiratory or pulmonary disease, diabetes mellitus, chronic heart failure, chronic liver disease, or chronic renal disease. This study-specific definition was broader than the current German STIKO definition of high-risk status and was used for subgroup stratification within the BUCOSS analyses.

Infection-related cost data were collected at baseline and during follow-up using dedicated cost forms. Baseline assessment captured episode-related costs already incurred during the 10 days before the index visit, including lost working hours, over-the-counter medication, travel expenses, and copayments for prescribed medication. Data were entered into an electronic data capture system by study staff. Healthcare utilization within the participating practice, including the index consultation, ARI-related diagnostic procedures and medications prescribed by the study practice, was documented by study staff, whereas patient-borne costs and resource use outside the participating practice were obtained by structured patient report using the same cost forms.

Only patients with RT-PCR-confirmed RSV infection entered follow-up and were contacted by telephone on Day 14 and, if not fully recovered, again on Day 28. Patients who had recovered sufficiently by Day 14 were considered to have completed follow-up for the RSV episode; no further practice-documented RSV-related healthcare utilization after Day 14 was recorded for these patients, and they were therefore not scheduled for an additional Day 28 assessment. During follow-up, persistent respiratory and systemic symptoms, time since symptom resolution where applicable, complications, and direct, indirect, and out-of-pocket costs were reassessed. Hospitalizations occurring within the 28-day observation period were documented separately but were excluded from the present analysis, which focused exclusively on outpatient management.

### Cost assessment

Costs were assessed from a societal perspective over 28 days. At baseline, selected cost components incurred during the preceding 10 days were additionally captured, including lost working hours, out-of-pocket costs, and copayments for prescribed medication. Prescription medication used before enrollment was not comprehensively assessed because prescriptions issued before presentation to participating practices were not systematically available and could only have been captured incompletely by patient self-report.

Only infection-related healthcare utilization, RSV-related diagnostic procedures, and symptom-related medications were included. Ongoing background medication for chronic conditions was considered only if prescribed specifically for infection-related symptom management.

Costs were grouped into direct medical costs borne by statutory health insurance, patient-borne out-of-pocket costs, and indirect costs, including productivity losses and informal care. Outpatient physician services were valued using the German Uniform Value Scale (EBM) [12], and prescribed medications were costed using pharmacy retail prices. Out-of-pocket costs included over-the-counter medication, transportation expenses, and statutory copayments for prescribed medication, which generally require 10% of the retail price, with a minimum of €5 and a maximum of €10 per package [13]. Travel by private car was valued at €0.30 per kilometer actually driven, in line with the flat-rate mileage allowance commonly applied in German tax guidance; where applicable, taxi costs were included at the full reported amount [14].

Indirect costs comprised patients’ work absenteeism (productivity losses) and time losses of accompanying persons (informal care). Informal care was valued using an opportunity cost approach based on the average net hourly wage in Germany [15, 16]; reported travel and waiting times were converted to a minute-based rate and monetized accordingly [17]. Productivity losses were valued using the human capital approach based on year-specific gross income data in line with German recommendations on health economic evaluation [18]. To account for annual changes in income levels, year-specific average gross employee income was converted into an hourly rate and applied to the reported number of working hours lost [19]; for participants in marginal employment (“Minijob”), the statutory minimum hourly wage in the respective year was used [20].

All cost components were valued using year-specific unit costs and wage levels. No additional inflation adjustment or discounting was applied. Further details on costing methods and unit cost sources are provided in the Supplementary Appendix S1.

### National burden extrapolation

To estimate the national outpatient burden of RSV infection in adults aged ≥ 60 years in Germany, two complementary extrapolation approaches were used. Because the participating practices were not selected through a formal population-based sampling strategy, these extrapolations were intended to provide approximate national burden estimates rather than precise nationally representative projections.

The primary approach applied the study-derived RSV incidence to the German population aged ≥ 60 years. This incidence estimate represents an approximation rather than an exact measure of the true national incidence of outpatient RSV infection. As a complementary approach, an ARE–prevalence-based extrapolation combined national surveillance-derived estimates of medically attended acute respiratory episodes (ARE) with the RSV prevalence observed in the study cohort. For economic extrapolation, both approaches were combined with the mean societal cost per outpatient RSV episode observed in the study cohort. Because RSV infections in temperate climates occur predominantly during winter epidemics, estimates are presented per RSV season. Further details are provided in the Supplementary Appendix S1.

### Statistical analysis

All analyses were exploratory. Baseline characteristics were summarized descriptively, and group comparisons used standard parametric or nonparametric methods, as appropriate. Prevalence and incidence estimates were calculated at state level and aggregated overall using prespecified weighting procedures. 95% confidence intervals were calculated using the Wilson score method. Symptom duration was analyzed using Kaplan-Meier methods. Because episode costs were expected to be right-skewed and partly zero-inflated, both mean and median costs were reported; mean costs were used for national burden estimation and extrapolation. Multivariable linear regression was used to assess the difference in productivity losses among employed patients along high-risk status, adjusting for age group and clinical presentation (URTI vs. LRTI). Further statistical details are provided in the Supplementary Appendix S1.

## Results

### Study population

Among 3,261 adults aged ≥ 60 years presenting with acute respiratory infection (ARI), 192 (5.9%) had PCR-confirmed RSV infection. The RSV prevalence was 5.49%, and the estimated RSV incidence was 1,030.6 cases per 100,000 population. After exclusion of four patients hospitalized at baseline, 188 non-hospitalized RSV-positive outpatients were included in the economic analysis. Among the 188 non-hospitalized RSV patients included in the economic analysis, 18 (9.6%) had one additional pathogen detected by multiplex RT-PCR. The most frequent co-pathogens were SARS-CoV-2 (*n* = 5) and human rhino-/enterovirus (*n* = 5), followed by human bocavirus (*n* = 2), coronavirus OC43 (*n* = 2), coronavirus NL63 (*n* = 1), influenza A (*n* = 1), influenza A/H1N1 (*n* = 1), and Bordetella pertussis (*n* = 1). In this final cohort, 113 patients (60.1%) had at least one underlying risk condition. Mean age was 71.6 years (SD 8.52), 57.4% were female, and mean ARI duration was 18.0 days (SD 8.46) (Table 1). Patients with underlying risk conditions were slightly older than those without risk conditions, whereas sex distribution (44.2% men among those with risk conditions vs. 40.0% among those without), duration of acute respiratory illness (17.9 vs. 18.1 days), and follow-up rates did not differ significantly between the two groups (Table 1). Day 14 follow-up was completed in 187/188 patients included in the outpatient cost analysis (99.5%). A Day 28 assessment was conducted in 95/188 patients (50.5%) according to the protocol-defined rule that patients were contacted again only if they had not recovered sufficiently by Day 14. Thus, the proportion of patients with a Day 28 assessment reflects extended follow-up due to incomplete recovery at Day 14 rather than loss to follow-up.

**Table 1:** Baseline characteristics of the RSV-positive outpatient population

Characteristic	Total (*N* = 188)	High-risk patients (*N* = 113)	Patients without high-risk status (*N* = 75)	*p*-value
*Age*,* years*				
Mean (SD)	71.6 (8.52)	72.7 (8.57)	70.0 (8.24)	0.035
Min–Max	60.0–93.4	60.0–93.4	60.0–91.1	
*Sex*				
Male, *n* (%)	80 (42.6)	50 (44.2)	30 (40.0)	0.670
Female, *n* (%)	108 (57.4)	63 (55.8)	45 (60.0)	
*ARI duration*,* days*				
Mean (SD)	18.0 (8.46)	17.9 (8.40)	18.1 (8.61)	0.889
Min–Max	1–39	1–39	3–36	
*Follow-up*				
14-day follow-up completed, *n* (%)	187 (99.5)	112 (99.1)	75 (100.0)	> 0.999
28-day follow-up completed, *n* (%)	95 (50.5)	59 (52.2)	36 (48.0)	0.677

*Legend*: Values are presented as mean (SD) or *n* (%). High-risk status was defined as the presence of at least one risk condition. Risk conditions included chronic respiratory diseases (e.g. asthma or chronic obstructive pulmonary disease), cardiovascular diseases, and other chronic comorbidities associated with increased vulnerability to respiratory infections

*Abbreviations*: *RSV* respiratory syncytial virus, *SD* standard deviation, *LABA* long-acting beta-agonist, *LAMA* long-acting muscarinic antagonist

Overall, 46 of 188 participants (24.5%) were still employed at the time of infection, of whom 28 (60.8%) were working full-time. Employment was less frequent among patients with underlying study-defined high-risk status than among those without risk conditions (22.1% vs. 34.7%), although this difference did not reach statistical significance (*p* = 0.084).

**Fig. 1:**
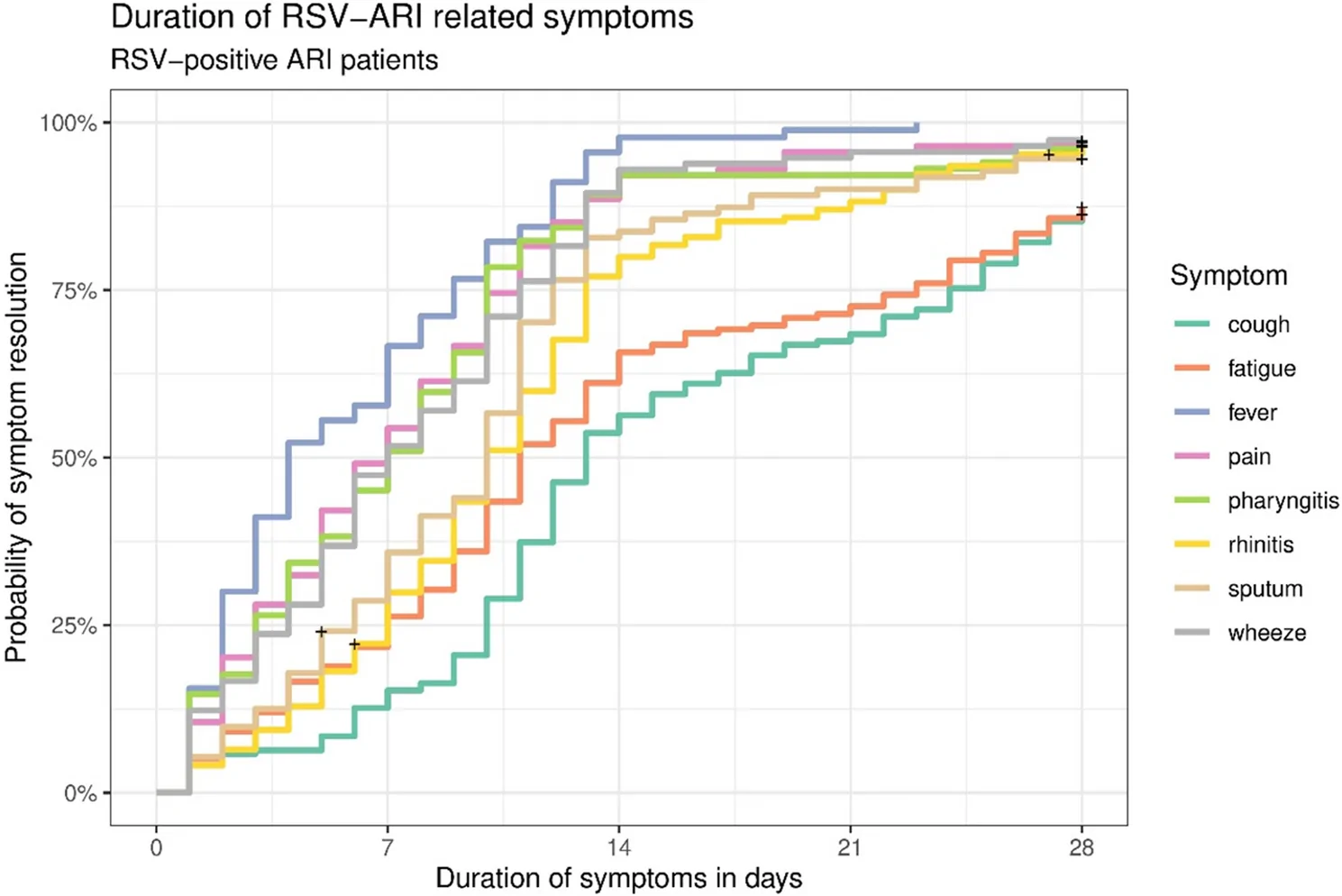
Kaplan-Meier estimates of time to symptom resolution over 28 days. Legend: Shown are Kaplan-Meier curves for time to resolution of individual symptoms during 28 days of follow-up in adults aged ≥ 60 years with PCR-confirmed RSV infection managed in the outpatient setting. The y-axis represents the cumulative probability of symptom resolution, and the x-axis the duration of symptoms in days. Curves are shown for cough, fatigue, fever, pain, pharyngitis, rhinitis, sputum production, and wheeze. Tick marks indicate censored observations, including symptoms that remained unresolved at the last available follow-up

Symptom-duration analyses support the use of a 28-day time horizon for the ambulatory cost analysis. By day 28, only a minority of patients still reported ongoing symptoms, most notably cough (13.7%) and fatigue (12.6%), while all other symptoms had declined to low rates (see Fig. 1). The figure also shows that cough and fatigue remained relatively frequent at day 14, supporting the relevance of follow-up beyond the initial visit. The most common complications were asthma or COPD exacerbation (16.1%) and bronchitis (14.6%).

### Costs

#### Healthcare utilization

Prescription medication was used by 126 (67.0%) RSV outpatients, over-the-counter medication by 90 (47.9%), and laboratory or other diagnostic services by 52 (27.7%). In addition to the mandatory baseline consultation, 39 (20.7%) patients had at least one additional practice visit during follow-up.

#### Cost distribution by high-risk status and detailed cost domains

Tables 2 and 3 summarize the overall cost distribution by high-risk status. Table 2 presents absolute costs (€), whereas Table 3 shows the percentage composition of total costs. Across all 188 RSV-positive cases, the mean total cost per episode was €510.0 (SD €742.58). Direct reimbursed costs averaged €161.3 and were the most stable cost component, whereas indirect costs averaged €331.8 (SD €746.75) and showed the greatest variability. Indirect costs were driven almost entirely by productivity loss (€330.8), while informal care was negligible (€1.0 per case overall).

**Table 2:** Summary of absolute costs by high-risk status in the RSV-positive outpatient cohort

Cost category	Overall mean (SD), €	High-risk patients, mean (SD), €	Patients without high-risk status, mean (SD), €	*p*-value
Total costs	510.0 (742.58)	418.6 (567.41)	647.7 (935.00)	0.993
Direct costs	161.3 (59.57)	171.2 (66.03)	146.4 (44.66)	< 0.001
Indirect costs, total	331.8 (746.75)	229.0 (568.38)	486.7 (937.95)	0.114
Productivity-loss costs	330.8 (746.64)	227.6 (567.81)	486.4 (938.05)	0.021
Informal care costs	1.0 (4.04)	1.4 (5.06)	0.3 (1.34)	0.115
Out-of-pocket costs	16.9 (22.25)	18.5 (25.08)	14.5 (17.04)	0.578

*Legend*: Indirect costs comprise productivity-loss costs and informal care costs

**Table 3:** Summary of the percentage distribution of cost components within total costs by high-risk status

Cost category	Overall mean (SD), %	High-risk patients, mean (SD), %	Patients without high-risk status, mean (SD), %	*p*-value
Direct costs	72.6 (34.14)	77.5 (30.93)	65.2 (37.48)	0.029
Indirect costs, total	20.6 (36.27)	15.5 (32.09)	28.5 (40.76)	0.092
Out-of-pocket costs	6.8 (8.37)	7.1 (8.64)	6.3 (8.00)	0.613

*Legend*: Percentages were calculated at the individual patient level as the share of each cost component in that patient’s total costs and were then averaged across patients; they therefore do not correspond to proportions derived from the overall mean absolute costs shown in Table 2

Mean total costs were higher in non-high-risk than in high-risk patients (€647.7 vs. €418.6), mainly because of higher productivity-loss costs (€486.4 vs. €227.6), whereas direct costs were higher in high-risk patients (€171.2 vs. €146.4). Mean out-of-pocket expenditure was €16.9 per patient, corresponding to 3.3% of mean total societal costs, and differed little between the groups. Of these patient-borne costs, over-the-counter medication in the 10 days before presentation accounted for €1,437.27 across the 188 RSV-positive patients, corresponding to €7.64 per patient (data not shown).

Table 3 shows a different pattern from Table 2 because it presents mean per-patient cost shares rather than proportions based on the overall mean absolute costs.

On average, 72.6% of total costs were attributable to direct costs, 20.6% to indirect costs, and 6.8% to out-of-pocket costs. High-risk patients meeting the study-defined high-risk criteria had a higher share of direct costs than non-high-risk patients (77.5% vs. 65.2%), whereas non-high-risk patients had a higher share of indirect costs (28.5% vs. 15.5%). Overall, high-risk cases showed a greater share of direct medical costs, whereas non-high-risk cases showed a greater share of productivity-loss costs.

Tables 4, 5 and 6 further examine the three cost domains that shaped the societal burden most clearly: reimbursed direct medical costs, productivity-loss costs, and total costs.

**Table 4:** Direct medical costs reimbursed by statutory health insurance

Group	*N*	Mean (SD), €	Median (IQR), €	Min–Max, €
Overall	188	161.30 (59.57)	143.95 (125.70, 172.51)	114–565
*Practice type*				
GP	118	161.27 (66.24)	142.22 (125.70, 168.00)	121–565
Pulmonologist	70	161.35 (46.65)	146.47 (125.70, 185.65)	114–343
*Age group*,* years*				
60–64	54	149.15 (49.28)	139.44 (120.69, 159.25)	114–418
65–69	33	155.97 (35.97)	145.62 (134.45, 172.53)	114–244
70–79	62	168.06 (73.95)	146.15 (125.70, 186.61)	119–565
80–89	36	170.25 (62.75)	153.05 (130.76, 185.58)	121–454
≥ 90	3	191.42 (48.39)	207.45 (137.04, 229.76)	137–230
*High-risk status*				
Yes	113	171.18 (66.03)	149.56 (133.38, 192.84)	115–565
No	75	146.42 (44.66)	134.45 (120.69, 154.50)	114–411
*Clinical presentation*				
URTI	72	142.35 (27.90)	135.20 (121.31, 150.19)	114–232
LRTI	116	173.06 (70.18)	151.30 (128.97, 186.61)	114–565
*RSV subtype*				
A	56	165.30 (54.00)	150.78 (130.49, 181.25)	121–454
B	130	159.92 (62.30)	140.07 (121.31, 171.87)	114–565
NA	2	138.82 (14.98)	138.82 (128.23, 149.41)	128–149

*Legend*: Direct medical costs reimbursed by statutory health insurance are shown for the overall cohort and stratified by practice type, age group, high-risk status, clinical presentation (URTI/LRTI), and RSV subtype.

Values are presented as mean (SD), median (IQR), and range (min–max). *URTI*  upper respiratory tract infection, *LRTI*  lower respiratory tract infection, *NA*  subtype not available

**Table 5:** Productivity-loss costs

Group	*N*	Patients with costs > 0, *n* (%)	Mean (SD), €	Median (IQR), €	Min–Max, €
Overall	188	46 (24.5)	330.84 (746.64)	0.00 (0.00, 0.00)	0–4,337
*Practice type*					
GP	118	27 (22.9)	332.43 (756.10)	0.00 (0.00, 0.00)	0–3,799
Pulmonologist	70	19 (27.1)	328.17 (735.81)	0.00 (0.00, 85.45)	0–4,337
*Age group*,* years*					
60–64	54	36 (66.7)	993.89 (1,067.66)	802.83 (0.00, 1,608.15)	0–4,337
65–69	33	7 (21.2)	181.48 (489.88)	0.00 (0.00, 0.00)	0–1,993
70–79	62	3 (4.8)	40.96 (189.79)	0.00 (0.00, 0.00)	0–1,087
80–89	36	0 (0.0)	0.00 (0.00)	0.00 (0.00, 0.00)	0–0
≥ 90	3	0 (0.0)	0.00 (0.00)	0.00 (0.00, 0.00)	0–0
*High-risk status*					
Yes	113	21 (18.6)	227.58 (567.81)	0.00 (0.00, 0.00)	0–2,650
No	75	25 (33.3)	486.43 (938.05)	0.00 (0.00, 695.24)	0–4,337
*Clinical presentation*					
URTI	72	21 (29.2)	409.49 (817.92)	0.00 (0.00, 396.69)	0–3,799
LRTI	116	25 (21.6)	282.03 (697.98)	0.00 (0.00, 0.00)	0–4,337
*RSV subtype*					
A	56	11 (19.6)	248.48 (606.17)	0.00 (0.00, 0.00)	0–3,178
B	130	35 (26.9)	371.41 (803.21)	0.00 (0.00, 223.47)	0–4,337
NA	2	0 (0.0)	0.00 (0.00)	0.00 (0.00, 0.00)	0–0

*Legend*: Productivity-loss costs excluding informal care are shown for the overall cohort and stratified by practice type, age group, high-risk status, clinical presentation (URTI/LRTI), and RSV subtype

Values are presented as mean (SD), median (IQR), and range (min–max). “Patients with costs > 0, *n* (%)” indicates the number and proportion of patients with productivity-loss costs greater than zero within the respective subgroup. Informal care was analyzed separately and is not included in this table. *URTI*  upper respiratory tract infection, *LRTI*  lower respiratory tract infection, *NA*  subtype not available

**Table 6:** Total societal costs

Group	*N*	Mean (SD), €	Median (IQR), €	Min–Max, €
Overall	188	510.01 (742.58)	186.06 (142.18, 364.98)	114–4,458
*Practice type*				
GP	118	511.91 (753.48)	181.53 (141.67, 368.99)	121–4,033
Pulmonologist	70	506.79 (729.22)	209.00 (142.21, 343.19)	114–4,458
*Age group*,* years*				
60–64	54	1,160.53 (1,063.05)	940.77 (194.29, 1,760.67)	114–4,458
65–69	33	355.47 (497.42)	177.49 (148.84, 234.20)	114–2,133
70–79	62	224.60 (203.87)	163.41 (132.42, 216.95)	121–1,262
80–89	36	193.07 (70.16)	166.96 (147.57, 217.29)	122–454
≥ 90	3	202.20 (59.29)	216.59 (137.04, 252.98)	137–253
*High-risk status*				
Yes	113	418.62 (567.41)	196.61 (147.00, 281.38)	121–2,818
No	75	647.69 (935.00)	183.77 (134.71, 815.93)	114–4,458
*Clinical presentation*				
URTI	72	564.78 (822.17)	161.41 (133.91, 622.93)	114–4,033
LRTI	116	476.01 (690.14)	197.80 (158.51, 323.36)	114–4,458
*RSV subtype*				
A	56	432.72 (600.14)	181.72 (141.60, 301.54)	121–3,299
B	130	548.64 (799.88)	189.33 (142.28, 477.17)	114–4,458
NA	2	162.96 (49.12)	162.96 (128.23, 197.69)	128–198

*Legend*: Total societal costs are shown for the overall cohort and stratified by practice type, age group, high-risk status, clinical presentation (URTI/LRTI), and RSV subtype

Values are presented as mean (SD), median (IQR), and range (min–max). Total costs comprise direct medical costs, indirect costs, and out-of-pocket costs. *URTI*  upper respiratory tract infection, *LRTI*  lower respiratory tract infection, *NA*  subtype not available

Reimbursed direct medical costs (Table 4) were comparatively homogeneous, with a mean of €161.30 (SD €59.57) and a median of €143.95 (IQR €125.70–172.51). All patients incurred direct costs. Costs differed little by practice type but increased with age, from €149.15 in patients aged 60–64 years to €170.25 in those aged 80–89 years; the very small 90 + group reached €191.42. Direct costs were higher in high-risk than in non-high-risk patients (€171.18 vs. €146.42) and higher in LRTI than in URTI (€173.06 vs. €142.35). Differences by RSV subtype were modest.

Productivity-loss costs excluding informal care (Table 5) showed a markedly right-skewed and zero-inflated distribution. Mean productivity-loss cost was €330.84 (SD €746.64), whereas the median was €0.00 (IQR €0.00–0.00), consistent with the strongly zero-inflated distribution; only 46 of 188 patients (24.5%) had any productivity-loss costs. Costs varied little across practice types but were highest among patients aged 60–64 years (€993.89; 66.7% with costs > 0), declined sharply in older age groups, and were absent among patients aged ≥ 80 years.

Across the full cohort, patients without high-risk status had higher mean productivity-loss costs than high-risk patients (€486.43 vs. €227.58) and more frequently incurred such costs (33.3% vs. 18.6%). Productivity-loss costs were also higher in URTI than in LRTI (€409.49 vs. €282.03) and in RSV B than in RSV A (€371.41 vs. €248.48).

In an analysis restricted to employed patients (*n* = 46; 21 high-risk and 25 without high-risk status), mean lost working hours were 47.5 h (SD 29.97) among employed high-risk patients and 55.0 h (SD 46.84) among employed patients without high-risk status (*p* = 0.519). Mean productivity-loss costs were €1,224.6 (SD €723.58) and €1,459.3 (SD €1,110.99), respectively (*p* = 0.394). In a multivariable linear model including high-risk status and adjusting for age group and clinical presentation (URTI vs. LRTI), adjusted mean lost working hours were 36.5 and 43.0 h, respectively (*p* = 0.596), and adjusted mean productivity-loss costs were €964.3 and €1,173.0, respectively (*p* = 0.476). Thus, among employed patients, differences in lost working hours and productivity-loss costs by high-risk status were not statistically significant.

Total costs (Table 6) reflected the same pattern. Mean total costs were €510.01 (SD €742.58) and the median was €186.06 (IQR €142.18–364.98), again indicating a right-skewed distribution. Costs varied little by practice type, but were by far highest in patients aged 60–64 years (mean €1,160.53, median €940.77) and much lower in older age groups. Mean total costs were lower in high-risk than in non-high-risk patients (€418.62 vs. €647.69), whereas median costs showed the opposite tendency (€196.61 vs. €183.77), suggesting that a subset of high-cost episodes in non-high-risk patients drove the higher mean. Mean total costs were somewhat higher in URTI than in LRTI (€564.78 vs. €476.01), whereas median total costs were higher in LRTI (€197.80 vs. €161.41), again indicating the influence of a smaller subgroup of high-cost URTI cases. Differences by RSV subtype were moderate, with higher mean total costs in RSV B than in RSV A (€548.64 vs. €432.72), while median values were similar.

In an exploratory analysis, costs and illness duration were compared between patients with and without co-pathogen detection. In non-parametric Wilcoxon rank-sum tests, total costs, direct costs, indirect costs, and out-of-pocket costs did not differ significantly between patients with and without co-pathogen detection. RSV-ARI duration was numerically longer in patients with co-pathogen detection than in those without co-pathogen detection (mean 19.9 vs. 17.8 days; median 22.5 vs. 14.0 days), but the corresponding Kaplan–Meier curves did not differ significantly in the log-rank test (*p* = 0.600).

### National burden

Applying the study-derived RSV incidence of 1,030.6 per 100,000 population to the 25,053,969 adults aged ≥ 60 years living in Germany in 2023 [21], and combining the resulting case estimate with the mean societal cost per outpatient RSV episode, yielded an estimated national burden of 258,206 medically attended outpatient RSV cases and €131.7 million per RSV season, including €41.6 million in direct medical costs, €85.7 million in indirect costs, and €4.4 million in out-of-pocket costs. The complementary ARE–prevalence-based extrapolation yielded 374,395 cases and €190.9 million per RSV season (see Supplementary Appendix S1, Section B, for details). Taken together, these two approaches should therefore be interpreted as providing an approximate range of medically attended outpatient RSV burden rather than a single nationally representative point estimate.

In an additional scenario analysis excluding indirect costs, the estimated national outpatient cost burden decreased to €46.0–66.7 million per RSV season, indicating that productivity losses substantially influenced the societal estimate but that a relevant cost burden remained when only direct reimbursed medical and patient-borne out-of-pocket costs were considered (see Supplementary Appendix S1, Section C, for details).

### Medication

Among the 188 non-hospitalized RSV patients, 126 (67.0%) received at least one physician-prescribed medication reimbursed by statutory health insurance (GKV). Total GKV-reimbursed medication costs amounted to €6,278.18. In addition, over-the-counter (OTC) medication purchased before presentation to the participating practice amounted to €1,437.27, corresponding to 22.9% of total GKV-reimbursed medication costs. Among treated patients, 35/126 (27.8%) received only other supportive medications, such as cough remedies or nasal drops, and none of the eight predefined medication classes. The costs of these other prescribed medications amounted to €1,223.19, corresponding to 24.2% of the costs of the eight predefined medication classes. The remaining €5,054.99 were attributable to the eight predefined medication classes, which were prescribed to 91/188 patients (48.4%).

To avoid double counting, patients with obstructive lung disease were classified into four mutually exclusive groups: asthma alone (*n* = 44), COPD alone (*n* = 25), both asthma and COPD (*n* = 5), and neither diagnosis (*n* = 114). As shown in Table 7, patients with asthma or COPD were prescribed systemic steroids (28/74; 37.8% vs. 2/114; 1.8%; OR 34.1; *p* < 0.001), anti-obstructive medications (18/74; 24.3% vs. 9/114; 7.9%; OR 3.75; *p* = 0.003), and antibiotics (31/74; 41.9% vs. 27/114; 23.7%; OR 2.32; *p* = 0.010) more often than patients without either diagnosis, whereas no clear difference was observed for inhaled steroids (2/74; 2.7% vs. 4/114; 3.5%). Notably, anti-obstructive medications were also prescribed to 9/114 patients (7.9%) without a documented diagnosis of asthma or COPD, and 14/114 (12.3%) without either diagnosis received at least one of the following three medication classes: systemic steroids, inhaled steroids, or anti-obstructive medications.

**Table 7:** Prescription of systemic steroids, inhaled steroids, and anti-obstructive medications by obstructive lung disease status

Diagnostic group	*n*	Systemic steroids	Inhaled steroids	Anti-obstructive medications	At least 1 of the 3 classes
Asthma alone	44	18/44 (40.9%)	0/44 (0.0%)	10/44 (22.7%)	24/44 (54.5%)
COPD alone	25	7/25 (28.0%)	1/25 (4.0%)	7/25 (28.0%)	11/25 (44.0%)
Both asthma and COPD	5	3/5 (60.0%)	1/5 (20.0%)	1/5 (20.0%)	3/5 (60.0%)
Neither diagnosis	114	2/114 (1.8%)	4/114 (3.5%)	9/114 (7.9%)	14/114 (12.3%)

*Legend*: Shown are the numbers and proportions of patients receiving systemic steroids, inhaled steroids, anti-obstructive medications, or at least one of these three medication classes across mutually exclusive diagnostic groups defined as asthma alone, COPD alone, both asthma and COPD, or neither diagnosis. Diagnostic groups were defined as mutually exclusive categories to avoid double counting

As shown in Table 8, antibiotics were the most frequently prescribed medication class (58/188; 30.9%), followed by systemic steroids (30/188; 16.0%) and anti-obstructive medications (27/188; 14.4%). The anti-obstructive category comprised combined anti-obstructive agents, single-agent LABA inhalers, and single-agent LAMA inhalers; because medication classes were not mutually exclusive, these accounted for 30 class entries in 27 individual patients. Although antibiotics were prescribed most frequently, combined anti-obstructive agents generated the highest class-specific total costs (€2,416.52) and the highest mean cost per treated patient (€105.07, SD €88.42). When anti-obstructive medications were considered jointly, total costs were €2,917.80, with a mean cost of €108.07 (SD €93.53) per treated patient, exceeding antibiotics (€1,385.25 total; €23.88 per treated patient) and systemic steroids (€415.07 total; €13.84 per treated patient). Overall, anti-obstructive medications accounted for the highest medication-related costs despite being prescribed less frequently than antibiotics.

**Table 8:** Frequency and costs of physician-prescribed medication classes among non-hospitalized RSV patients

Medication class	All RSV patients (*n* = 188)	Treated patients (*n* = 126)	Total costs, €	Mean cost per treated patient, €
Combined anti-obstructive agents	23/188 (12.2%)	23/126 (18.3%)	2,416.52	105.07 (SD 88.42)
LABA	3/188 (1.6%)	3/126 (2.4%)	129.64	43.21 (SD 32.71)
LAMA	4/188 (2.1%)	4/126 (3.2%)	371.64	92.91 (SD 76.60)
Antibiotics	58/188 (30.9%)	58/126 (46.0%)	1,385.25	23.88 (SD 12.59)
Systemic steroids	30/188 (16.0%)	30/126 (23.8%)	415.07	13.84 (SD 3.06)
Inhaled steroids	6/188 (3.2%)	6/126 (4.8%)	116.32	19.39 (SD 10.45)
NSAIDs	9/188 (4.8%)	9/126 (7.1%)	116.23	12.91 (SD 1.35)
Antipyretics	9/188 (4.8%)	9/126 (7.1%)	104.32	11.59 (SD 5.81)
Total across 8 medication classes			5,054.99	

*Legend*: Shown are prescription frequencies and costs for the eight predefined physician-prescribed medication classes in the 188 non-hospitalized RSV patients and in the subgroup of 126 patients receiving any physician-prescribed medication. Total costs are reported per medication class, and mean costs refer to treated patients within the respective class. Note: Medication classes were not mutually exclusive; therefore, patient counts across classes sum to more than the number of treated patients. The anti-obstructive category comprised combined anti-obstructive agents, single-agent LABA inhalers, and single-agent LAMA inhalers

## Discussion

Evidence on the outpatient costs of RSV infection in older adults remains limited and heterogeneous. Among the few available studies, the Japanese claims-based analysis by Igarashi et al. [22] reported comparatively low outpatient medical costs of ¥11,306.6 (approximately US$86.3) for non-hospitalized patients with an emergency visit and ¥6,660.6 (approximately US$50.8) for those without an emergency visit. However, these estimates were based on very small ambulatory subgroups and did not include prescription dispensing or pharmacy fees. Two recent German claims-based studies by Marijic et al. [8] and Huebbe et al. [7] also reported lower outpatient costs than those observed in our study. Marijic et al. [8] reported mean excess outpatient costs of €116 per confirmed RSV episode in adults aged ≥ 60 years, whereas Huebbe et al. [7] reported mean outpatient costs of €127 per RSV-specific episode, with higher excess costs in adults aged 60–74 years. Their handling of medication costs differed: Marijic et al. [8] restricted the analysis to relevant ATC-coded medications and compared cases with matched controls, whereas Huebbe et al. included all prescribed drugs within a 90-day observation period. Consequently, Huebbe’s outpatient drug costs may partly reflect background medication use in older multimorbid adults, whereas Marijic’s estimates are more tightly confined to infection-attributable medication costs.

In contrast to the small prospective study by Mao et al. [23], which included only 36 patients and did not document productivity losses, the Dutch–Italian primary care study by Hak et al. [24] was the first sufficiently large prospective investigation to demonstrate a measurable socioeconomic burden of ambulatory RSV infection in older adults. It shares important methodological features with our study, including PCR-confirmed RSV, patient-level questionnaire data, and prospective follow-up over a defined illness episode. Nevertheless, mean costs per episode were lower than in our cohort, at €78.1 from the healthcare system perspective and €279.7 from the societal perspective. This likely reflects more comprehensive cost ascertainment in our study. Hak et al. did not include diagnostic costs, whereas our analysis incorporated systematic multiplex PCR testing, distinguished reimbursed from patient-borne expenditures, and captured work absenteeism in greater detail. Our findings therefore extend the available evidence by providing a more comprehensive and Germany-specific estimate of the societal burden of ambulatory RSV infection in older adults.

Case ascertainment also differed fundamentally from claims-based analyses. In our cohort, all included cases were verified by multiplex PCR, permitting cost estimates to be linked to laboratory-confirmed RSV episodes. By contrast, claims-based studies identify RSV through ICD-10 codes in administrative data, without direct access to laboratory results. It therefore remains uncertain whether virological testing was performed, whether associated diagnostic costs were captured, or whether coded cases truly represented laboratory-confirmed RSV rather than another respiratory infection managed under routine care conditions.

The observed cost structure is particularly informative. Informal care contributed negligibly to total societal costs, whereas productivity-loss costs accounted for almost two thirds of the total burden. Thus, the societal burden of outpatient RSV in older adults was driven primarily by work-related losses rather than informal caregiving. Employment status is central to interpreting this pattern. In Hak et al. [24], only 14 of 86 participants (approximately 16%) were employed, largely reflecting the high proportion of individuals aged ≥ 75 years. In our cohort, 24.5% remained employed, including 22.1% of high-risk patients and 34.7% of non-high-risk patients. When productivity-loss costs were averaged across all patients, including those with no productivity-loss costs, mean productivity-loss costs were higher in patients without high-risk status. However, in the analyses restricted to employed patients, differences in lost working hours and productivity-loss costs by high-risk status were not statistically significant; this remained the case after adjustment for age group and clinical presentation. These findings suggest that the higher productivity-loss costs observed in patients without high-risk status were mainly driven by the higher proportion of employed individuals in this group rather than by clear evidence of greater work loss among employed patients without high-risk status.

A further methodological difference between our study and claims-based analyses concerns the measurement of productivity losses. In our study, these were recorded prospectively through detailed patient questionnaires, allowing short-term and partial work loss to be captured. By contrast, claims-based analyses depend on formally documented sick-leave days in administrative records. In Germany, sick-leave certificates are usually issued only from the third day of illness onwards, and claims data typically record complete days rather than hours. Short-term or partial work loss may therefore remain undocumented. This interpretation is supported by previous German routine-data studies: Huebbe et al. [7] reported mean outpatient productivity loss of only 0.76 days in adults aged 60–74 years and 0 days in those aged ≥ 75 years, while Marijic et al. [8] identified sick-leave certificates in only 3.7% of confirmed RSV cases aged ≥ 60 years. Against this background, the markedly higher proportion of patients with lost working hours in our cohort (24.5%) suggest that routine administrative data may underestimate the true productivity burden of outpatient RSV infection. Consistent with this, total societal costs were highest in the 60–69-year age group, corresponding to the youngest and most economically active subgroup.

Prescription of anti-obstructive respiratory medication was analyzed as an additional outcome of interest. As expected, patients with asthma or COPD were more likely than those without either diagnosis to receive systemic corticosteroids, anti-obstructive medication, and antibiotics, consistent with earlier reports describing bronchodilator and corticosteroid use in adults with RSV, particularly in those with asthma, COPD, bronchospasm, or wheeze [25–28]. However, anti-obstructive treatment was not restricted to patients with documented obstructive lung disease, suggesting that acute RSV-related respiratory symptoms may also prompt bronchodilator therapy in the absence of established chronic airway obstruction. Although antibiotics were prescribed most frequently, anti-obstructive agents accounted for the largest share of medication-related costs, indicating that treatment of obstructive respiratory symptoms was a key driver of expenditure. Because previous cohort studies have not shown clear outcome benefits of corticosteroids in hospitalized adults with RSV [26, 27], these prescribing patterns are more likely to reflect symptomatic management of obstructive features than RSV-specific therapy.

Our findings also highlight the relevance of patient-borne medication costs. OTC expenditure in the 10 days before presentation amounted to €1,437.27 across the 188 RSV-positive patients (€7.64 per patient), representing 22.9% of total reimbursed medication costs (€6,278.18) and 28.4% when compared with the eight predefined medication classes (€5,054.99). In addition, prescribed supportive medications outside these eight classes accounted for a further €1,223.19, equivalent to 24.2% of the costs of the predefined classes. Thus, both OTC medication and additional physician-prescribed supportive medication contributed materially to the overall medication burden. These expenditures are not fully captured in routine claims data, suggesting that payer-based analyses may underestimate the true pharmaceutical burden of outpatient RSV infection in older adults.

This study has several strengths. It is among the very few prospective investigations to assess the societal cost of ambulatory RSV infection in older adults using PCR-confirmed cases and provides detailed patient-level data across major cost components. Unlike claims-based analyses, it directly captured productivity losses, informal care, and out-of-pocket expenditures in addition to reimbursed healthcare costs. The multicenter design and inclusion of three consecutive RSV seasons further strengthen the robustness of the findings.

Several limitations should also be considered. First, the study did not include a matched control group of ARI patients without RSV and therefore does not allow estimation of RSV-attributable excess costs relative to other respiratory infections. However, the primary aim was not causal attribution, but comprehensive measurement of outpatient costs associated with PCR-confirmed RSV infection in routine care. Second, some economic data were based on patient self-report and may be subject to recall bias, although the observation period was short. Third, although the final study network covered general practitioner and pulmonary specialist practices in metropolitan and rural settings across eight federal states, participating practices were not selected through a formal population-based random sampling procedure; therefore, strict national representativeness of the enrolled outpatient population cannot be assumed. Accordingly, the national case and cost estimates should therefore be interpreted as approximate extrapolations of medically attended outpatient RSV burden rather than precise nationally representative projections. Fourth, because the study included only medically attended outpatient cases and did not capture community RSV infections without healthcare contact, the overall outpatient societal burden is likely underestimated. Finally, although the 28-day follow-up period corresponds well to the acute clinical course of RSV infection, some residual burden and downstream morbidity beyond this time window could not be captured and therefore cannot be excluded.

Despite these limitations, this study provides important new evidence on the economic burden of RSV infection in older adults. Outpatient RSV episodes generated relevant costs not only within the healthcare system, but also from a broader societal perspective. Although the cost per outpatient episode is lower than that of hospitalization, the much larger number of ambulatory cases may result in a substantial cumulative burden at population level. Clinical trials of licensed RSV vaccines have demonstrated high efficacy against clinically relevant RSV disease in adults aged ≥ 60 years, including medically attended illness and RSV-associated lower respiratory tract disease [29–32]. Vaccination may therefore reduce not only severe outcomes and hospitalizations, but also outpatient healthcare utilization and related societal costs.

In conclusion, the outpatient economic burden of RSV infection in older adults appears higher than previously suggested by payer-based analyses or studies with less comprehensive cost capture. Depending on the extrapolation framework used, the two approaches yielded approximate estimates of 258,000 to 374,000 medically attended outpatient RSV cases per season among adults aged ≥ 60 years in Germany. Taken together, our findings underscore that RSV in older adults is not merely a hospital-based problem, but also a substantial ambulatory and societal burden, with important implications for health-economic evaluations, vaccination policy, and cost-effectiveness analyses of RSV prevention strategies in aging populations.

## Supplementary Information

Supplementary Material 1.

Supplementary Material 2.

## Data Availability

All relevant aggregated data supporting the conclusions of this article are included within the article and its Supplementary Information file. Individual-level deidentified participant data, including data dictionaries, will not be shared because of data protection and privacy restrictions.

## Abbreviations

ARI: Acute respiratory infection
ARE: Acute respiratory episode
BUCOSS: BUrden of Disease and COst of illneSS
CAPNETZ: Competence Network for Community-Acquired Pneumonia
COPD: Chronic obstructive pulmonary disease
EBM: Einheitlicher Bewertungsmaßstab
GKV: Gesetzliche Krankenversicherung
GP: General practitioner
LABA: Long-acting beta-agonist
LAMA: Long-acting muscarinic antagonist
LRTI: Lower respiratory tract infection
OTC: Over-the-counter
PCR: Polymerase chain reaction
RSV: Respiratory syncytial virus
RT-PCR: Reverse transcription polymerase chain reaction
STIKO: Standing Committee on Vaccination at the Robert Koch Institute (Ständige Impfkommission am Robert Koch-Institut)
URTI: Upper respiratory tract infection
