# Supplementary material for: The outpatient economic burden of respiratory syncytial virus infection in adults aged ≥ 60 years in Germany: results from the BUCOSS study
Source: Respir Res. 2026 Aug 14;27:344. doi: 10.1186/s12931-026-03866-1 (PMC13546509; doi:10.1186/s12931-026-03866-1)
Supplement: Supplementary file 1 [file 12931_2026_3866_MOESM1_ESM.docx]

**Supplementary Appendix S1. Supplementary methods, supplementary analyses and detailed worked calculations**

**A. Study procedures and follow-up**

Patients aged ≥60 years presenting with acute respiratory infection (ARI) in participating outpatient practices were enrolled after written informed consent. The participating practices were located in eight federal states: Baden-Württemberg, Bavaria, Berlin, Brandenburg, Hesse, Lower Saxony, North Rhine-Westphalia, and Saxony-Anhalt. At baseline (Day 0), demographic and clinical data, including medical history, comorbidities, employment status, and health-related quality of life (HRQoL), were collected. In addition, a nasopharyngeal swab was obtained for multiplex RT-PCR testing for RSV A/B and a broader respiratory pathogen panel comprising SARS-CoV-2, human bocavirus, seasonal coronaviruses 229E, HKU1, NL63 and OC43, influenza viruses A, A/H1N1, A/H3N2 and B, human metapneumovirus, parainfluenza viruses 1–4, human rhino-/enterovirus, Bordetella pertussis, Chlamydia pneumoniae, Legionella pneumophila, and Mycoplasma pneumoniae.

Infection-related cost data were collected at baseline and during follow-up using dedicated cost forms. Baseline cost assessment captured episode-related costs already incurred during the 10 days before the index practice visit, including lost working hours, over-the-counter medication, travel expenses, and copayments for prescribed medication. Data were entered into an electronic data capture system by study staff. Healthcare utilization within the participating practice, including the index consultation, ARI-related diagnostic procedures and medications prescribed by the study practice, was documented by study staff, whereas patient-borne costs and resource use outside the participating practice were obtained by structured patient report using the same cost forms. The English translation of these forms is provided as Supplementary Appendix S2.

Only patients with RT-PCR-confirmed RSV infection entered follow-up and were contacted by telephone on Day 14 and, if not fully recovered, again on Day 28. Patients who had recovered sufficiently by Day 14 were considered to have completed follow-up for the RSV episode; no further practice-documented RSV-related healthcare utilization after Day 14 was recorded for these patients, and they were therefore not scheduled for an additional Day 28 assessment. During follow-up, persistent respiratory and systemic symptoms, time since symptom resolution where applicable, complications, and direct, indirect, and out-of-pocket costs were reassessed. In addition, HRQoL was evaluated as a secondary endpoint using the EQ-5D-3L at baseline (Day 0), Day 14, and Day 28, if applicable. Hospitalizations occurring within the 28-day observation period were documented separately but were excluded from the present analysis, which focused exclusively on outpatient management.

*Cost perspective and general costing principles*

Costs were assessed from a societal perspective over a 28-day observation period. At baseline, selected cost components incurred during the 10 days preceding the index visit were additionally captured. For this pre-index period, lost working hours, out-of-pocket costs, and copayments for prescribed medication were recorded. Prescription medication used before enrollment was not comprehensively assessed because prescriptions issued before presentation to participating practices were not systematically available and could only have been captured incompletely by patient self-report.

To avoid artificial cost inflation, only symptom-related medications, RSV-related diagnostic procedures, and other infection-related healthcare utilization were included. Medications, investigations, and healthcare use unrelated to the acute respiratory infection were excluded irrespective of underlying comorbidities. In particular, ongoing background medication for chronic conditions was not included unless prescribed specifically for infection-related symptom management.

Included cost items were grouped into: (1) direct medical costs borne by statutory health insurance (SHI), including laboratory costs, outpatient physician fees, prescribed medication, and medical procedures; (2) out-of-pocket costs borne by patients themselves, including direct medical and direct non-medical expenses; and (3) indirect costs, including productivity losses and informal care.

*Direct medical costs borne by statutory health insurance*

Outpatient physician services were valued using the quarterly versions of the German Uniform Value Scale (Einheitlicher Bewertungsmaßstab, EBM) published by the National Association of Statutory Health Insurance Physicians (Kassenärztliche Bundesvereinigung, KBV) and applicable during the respective observation period from 2022 to 2025 ^1^. Costs were calculated by applying the fixed euro amounts or point values specified for the relevant services.

Drug costs were calculated using pharmacy retail prices (Apothekenverkaufspreise, AVP) according to prescribed package sizes, derived from the *Rote Liste®* or, where not available, from pharmacy software used for routine dispensing, valid at the time of prescription. Drug costs therefore reflect retail prices charged in routine outpatient care in Germany.

*Out-of-pocket costs*

Out-of-pocket (OoP) costs were defined as direct medical and direct non-medical expenses paid by patients themselves and not reimbursed by SHI. OoP costs comprised over-the-counter medication purchased during the pre-visit period, transportation expenses, and copayments for prescribed medication reimbursed by SHI. Patient copayments for prescribed medicines were calculated according to German statutory copayment rules (§ 61 SGB V), which generally require 10% of the retail price, with a minimum of €5 and a maximum of €10 per package^2^.

Time losses related to transportation by accompanying persons were not classified as out-of-pocket costs because they do not represent direct monetary payments, but rather indirect opportunity costs.

Travel by private car was valued at €0.30 per kilometer actually driven, in line with the flat-rate mileage allowance commonly applied in German tax guidance for business travel reimbursement across all study seasons^3^. Where applicable, taxi costs were included at the full reported amount and rounded to the nearest euro.

*Indirect costs*

Indirect costs reflected productivity losses and time-related opportunity costs attributable to the RSV episode. These included patients’ work absenteeism, measured as hours of work incapacity, as well as time losses incurred by accompanying persons who provided informal support, such as transporting patients to and from participating practices.

*Informal care*

Time losses of accompanying persons transporting patients to medical visits were classified as indirect costs rather than out-of-pocket expenditures, as they do not constitute direct monetary payments^4,5^. These time costs were valued using an opportunity cost approach based on the average net hourly wage in Germany^6^. Reported travel and waiting time, where applicable, were multiplied by the corresponding minute-based rate to derive monetary time costs. This approach was considered appropriate because accompanying persons may be retired, unemployed, or otherwise not engaged in paid work; therefore, their time losses do not necessarily reflect actual labor market productivity.

*Productivity losses*

Productivity losses were valued using the human capital approach and labor market prices, in line with the German recommendations on health economic evaluation (Hanover Consensus)^7^. To account for annual changes in income levels, year-specific average gross employee income was converted into a daily value based on 365 days and subsequently into an hourly rate, which was then applied to the reported number of working hours lost^8^.

For participants in marginal employment (“Minijob”), productivity losses were not valued using the monthly earnings threshold as a lump-sum amount, because short-term sickness absence would not be expected to result in loss of the full monthly amount. Instead, the statutory minimum hourly wage applicable in the respective study year was used^9^. This hourly rate was multiplied by the reported lost working hours, capped at 10 hours per week, which was the assumed maximum weekly working time for marginal employment.

*Cost valuation, inflation adjustment, and discounting*

All cost components were valued using year-specific unit costs and wage levels corresponding to the respective year of data collection. Reimbursement rates, transportation-related unit costs, and productivity losses were therefore calculated using contemporaneous price and income levels applicable in each calendar year. As these year-specific valuations already reflect annual changes in prices and wages, no additional inflation adjustment was applied. Reported costs therefore represent nominal values in the respective observation years. Given the short observation period and the episode-based nature of the analysis, no discounting was performed.

*Statistical analysis*

All analyses were exploratory. Baseline characteristics were summarized separately for participants with RSV-ARI and those with ARI due to other respiratory pathogens. Continuous variables are presented as mean and standard deviation (SD), and categorical variables as counts and percentages. Between-group comparisons used Wilcoxon rank-sum tests for continuous variables and chi-square tests or Fisher exact tests, as appropriate, for categorical variables. The study was designed as a descriptive study, and uncertainty for selected estimates was expressed using 95% confidence intervals (95% CIs).

Pathogen-specific prevalence and incidence were estimated at the level of the federal states, rather than at the level of individual study sites, in order to account for regional differences in recruitment intensity and site participation.

For prevalence, the analysis was based on enrolled participants with a valid RT-PCR result. For each federal state i, RSV prevalence was defined as

$$\pi_{\mathrm{RSV},i}=\frac{N_{\mathrm{RSV},i}}{N_{\mathrm{valid},i}}$$

where $N_{\mathrm{RSV},i}$ denotes the number of enrolled participants with RT-PCR-confirmed RSV in state $i$ and $N_{\mathrm{valid},i}$ is the number of enrolled participants with a valid RT-PCR result in that federal state. The overall RSV prevalence was then calculated as a weighted estimate across federal states. To account for differences between federal states in the proportion of ARI patients who were enrolled and tested, the observed RSV positivity among PCR-tested participants was scaled to the total ARI case load reported by participating practices in the same federal state. The weight for state $i$ was defined as

$w_{i}=\frac{N_{\mathrm{ARI},i}}{N_{\mathrm{valid},i}}$*,*

where $N_{\text{ARI},i}$ is the total number of ARI patients reported by participating practices in that state during the observation period. The overall prevalence was calculated as

$\pi_{\text{RSV}}=\frac{\sum_{i=1}^{m} w_{i}N_{\text{RSV},i}}{\sum_{i=1}^{m} w_{i}N_{\text{valid},i}}$*.*

This approach estimates the proportion of RSV-positive cases among all ARI patients represented by the participating practices, while adjusting for differences between federal states in the ratio of PCR-tested study participants to all ARI patients seen in routine care.

Incidence was estimated in two steps. First, the number of RSV-positive ARI cases was estimated for each federal state by scaling the observed number of RSV-positive study participants to the total number of ARI patients reported by the participating practices in that state. Specifically,

$$N_{\mathrm{RSV}\text{(ARI)},i}=\frac{N_{\mathrm{RSV},i}}{N_{\mathrm{valid},i}/N_{\mathrm{ARI},i}}=N_{\mathrm{RSV},i}\times\frac{N_{\mathrm{ARI},i}}{N_{\mathrm{valid},i}}.$$

This means that the RSV positivity observed among PCR-tested study participants was applied to the full ARI population reported by the participating practices in the same federal state. In other words, the clinical cohort was used to estimate the RSV proportion among ARI cases, and this proportion was then extrapolated to all ARI patients seen by the participating practices within the same federal state.

Second, the estimated number of RSV-positive ARI cases was related to the corresponding population denominator. For each federal state, incidence was defined as

$\mathrm{inc}_{\mathrm{RSV},i}=\frac{N_{RSV(ARI),i}}{N_{\mathrm{TC},i}}$,

where $N_{\text{TC},i}$ is the number of treatment certificates for patients aged ≥60 years in state $i$, averaged over the two quarters covering the respective observation period. The quotient was multiplied by 100,000; incidence was therefore reported as RSV-positive ARI cases per 100,000 persons aged ≥60 years.

The overall incidence was obtained by weighted aggregation across federal states. For this purpose, weighted sums of the estimated RSV-positive ARI cases and the corresponding treatment-certificate denominators were used. The state-specific incidence weights reflected the proportion of observation weeks contributed by participating sites in each federal state relative to the total number of weeks in the observation period. This weighting was applied to account for differences in the duration and intensity of site participation across federal states and seasons. The overall incidence was therefore calculated from the weighted total number of estimated RSV-positive ARI cases divided by the weighted total number of treatment certificates and reported per 100,000 persons aged ≥60 years.

This incidence estimation assumes that, within each federal state, RSV positivity among enrolled participants with valid RT-PCR results approximates RSV positivity among all ARI patients reported by the participating practices during the same observation period. It further assumes that the reported ARI case load adequately reflects the ARI patients seen in routine care at participating practices and that the treatment-certificate denominator provides an appropriate outpatient care denominator for adults aged ≥60 years. The weighting procedure was used to account for differences in the duration and intensity of site participation across federal states and seasons.

Symptom duration up to 28 days was available for patients with RSV-ARI only and was analyzed using Kaplan–Meier methods to estimate symptom-specific time-to-resolution curves; symptoms persisting at Day 28 were censored.

Cost data were analyzed from a societal perspective. Continuous cost variables were summarized using mean and SD as well as median and interquartile range (IQR), as appropriate, and categorical variables as absolute and relative frequencies. As episode costs were expected to show a right-skewed and partly zero-inflated distribution, both mean and median costs were prespecified for reporting. Mean costs were considered the primary metric for estimating the overall societal burden, for national extrapolation, and for cross-study comparison, as previous outpatient RSV cost studies have likewise reported mean-based episode costs. Median costs were used as a complementary measure to describe the typical patient-level episode. This distinction was considered particularly relevant for productivity-loss costs, for which the median may be zero despite a substantial contribution to the overall societal burden within the economically active subgroup.

For subgroup analyses, descriptive statistics were reported stratified by high-risk status, age group, and clinical presentation (URTI/LRTI). Given the exploratory nature of these analyses, subgroup comparisons were interpreted descriptively. No formal adjustment for multiple testing was applied, as subgroup analyses were exploratory.

To further explore productivity losses by high-risk status, an additional analysis restricted to employed patients was performed. Unadjusted group comparisons were supplemented by a multivariable linear model including high-risk status and adjusting for age group and clinical presentation (URTI vs LRTI).

An exploratory comparison by co-pathogen status was also performed. Costs were compared between patients with and without co-pathogen detection using Wilcoxon rank-sum tests. RSV-ARI duration by co-pathogen status was compared using Kaplan-Meier curves and the log-rank test.

*National burden extrapolation*

To approximate the potential national outpatient burden of RSV infection in older adults in Germany, two complementary extrapolation approaches were explored.

The primary approach was an incidence-based extrapolation. In this approach, the study-derived RSV incidence was applied to the German population aged ≥60 years using official population statistics from the German Federal Statistical Office as the denominator. The resulting number of estimated outpatient RSV cases was then combined with the mean societal cost per outpatient RSV episode observed in the cost cohort.

As a complementary approach, an ARE–prevalence-based extrapolation was performed. This approach combined national surveillance-derived estimates of medically attended acute respiratory episodes (ARE) among adults aged ≥60 years with the RSV prevalences observed in the study cohort. Because patients in the study were recruited from outpatient care after presenting with respiratory illness and were subsequently tested for RSV, this multiplier approach mirrors the clinical recruitment pathway.

For health-economic interpretation, both extrapolation approaches were combined with the mean societal cost per outpatient RSV episode observed in the cost cohort. Because RSV infections in temperate climates occur predominantly during winter epidemics, the resulting estimates are presented per RSV season rather than as annual costs.

To ensure transparency and facilitate reproducibility, detailed worked calculations for the national burden extrapolation are provided in Section B of the Supplement. These include the underlying data sources, formulas, seasonal population denominators, and the step-by-step derivation of the incidence-based and ARE–prevalence-based estimates.

**B. Detailed worked calculations for the national burden extrapolation**

Data sources

Weekly age-specific consultation incidence rates for acute respiratory illness (ARE) were obtained from the AGI sentinel surveillance dataset *ARE-Konsultationsinzidenz* published by the Robert Koch Institute (RKI)^10^. Population denominators for adults aged ≥60 years were obtained from the German Federal Statistical Office (Destatis), based on the official population update by age^11,12^. The October-to-April seasonal observation periods were aligned with the study observation windows described in the BUCOSS report^13^. Study-derived RSV prevalence and incidence estimates were taken from the BUCOSS study analyses.

The following study-derived inputs were used:

- overall RSV incidence: 1,030.6 per 100,000
- season-specific RSV incidence estimates: 901.4, 1,216.4, and 1,145.7 per 100,000
- season-specific RSV prevalences: 12.0%, 3.7%, and 5.5%
- mean societal cost per outpatient RSV episode: €510.0

*1. Incidence-based extrapolation*

In the primary approach, the study-derived RSV incidence was directly applied to the German population aged ≥60 years.

Formula

N_RSV = Incidence_RSV × Population_60+ / 100,000

Using the overall incidence estimate of 1,030.6 per 100,000 and the German population aged ≥60 years in 2023 (25,053,969)^11,12^, the estimated number of medically attended outpatient RSV cases was:

N_RSV = 1,030.6 × 25,053,969 / 100,000
N_RSV = 258,206

Thus, the incidence-based approach yielded 258,206 medically attended outpatient RSV cases per RSV season.

For economic extrapolation, this case number was multiplied by the mean societal cost per outpatient RSV episode:

258,206 × €510.0 = €131,685,060

This corresponds to an estimated societal burden of €131.7 million per RSV season.

*2. ARE–prevalence-based extrapolation*

Step 1: Definition of seasonal windows

Although the study observation period was defined on a calendar basis from October to April^13^, the AGI-based extrapolation was operationalized using complete surveillance weeks from calendar week 40 to calendar week 17 in all three seasons^10^. This ensured methodological consistency across seasons and alignment with routine AGI/RKI weekly reporting.

Accordingly, the following seasonal windows were used:

- 2022/23: CW40/2022 to CW17/2023
- 2023/24: CW40/2023 to CW17/2024
- 2024/25: CW40/2024 to CW17/2025

Step 2: Weekly age-specific ARE consultation incidence values

For each calendar week within the relevant seasonal window, the weekly national ARE consultation incidence for adults aged ≥60 years was extracted from the AGI dataset^10^. Each weekly value is expressed as the number of outpatient consultations for acute respiratory illness per 100,000 population aged ≥60 years.

Step 3: Summation of weekly incidence values

For each season, the weekly ARE consultation incidence values were summed across the defined seasonal window^10^:

Inc_season = Σ Inc_week

This yielded cumulative seasonal ARE consultation incidences of:

- 22,026.0 per 100,000 in 2022/23
- 19,167.3 per 100,000 in 2023/24
- 20,977.8 per 100,000 in 2024/25

Step 4: Population denominators

Season-specific population denominators were taken from the official population count as of 31 December of the respective year, because this standardized reference date falls within each October-to-April observation period and provides an appropriate approximation of the mid-season population at risk^11,12^.

The population denominators used were:

- 24,648,078 (31 Dec 2022)
- 25,053,969 (31 Dec 2023)
- 25,483,967 (31 Dec 2024)

Step 5: Conversion into absolute numbers of ARE episodes

The cumulative seasonal incidence per 100,000 population was converted into an absolute number of medically attended ARE episodes using the corresponding season-specific population denominator^10-12^:

N_ARE = Inc_season × Population_60+ / 100,000

This resulted in:

2022/23
22,026.0 × 24,648,078 / 100,000 = 5,428,986

2023/24
19,167.3 × 25,053,969 / 100,000 = 4,802,094

2024/25
20,977.8 × 25,483,967 / 100,000 = 5,346,027

Total across all three seasons:

5,428,986 + 4,802,094 + 5,346,027 = 15,577,107

Thus, the AGI/Destatis-based calculation yielded 15,577,107 medically attended ARE episodes among adults aged ≥60 years across the three study seasons.

Step 6: Derivation of RSV cases from ARE episodes

In a second step, the season-specific numbers of medically attended ARE episodes were multiplied by the corresponding study-derived RSV prevalences:

N_RSV = N_ARE × Prev_RSV

Using RSV prevalences of 12.0%, 3.7%, and 5.5%, the resulting estimates were:

2022/23
5,428,986 × 0.12 = 651,478

2023/24
4,802,094 × 0.037 = 177,677

2024/25
5,346,027 × 0.055 = 294,031

Total across all three seasons:

651,478 + 177,677 + 294,031 = 1,123,186

Average per season:

1,123,186 / 3 = 374,395

Thus, the ARE–prevalence-based approach yielded 374,395 medically attended outpatient RSV cases per RSV season.

For economic extrapolation:

374,395 × €510.0 = €190,941,450

This corresponds to an estimated societal burden of €190.9 million per RSV season.

3. *Summary of both extrapolation approaches*

The incidence-based approach yielded 258,206 medically attended outpatient RSV cases per season, corresponding to an estimated societal burden of €131.7 million per RSV season.

The ARE–prevalence-based approach yielded 374,395 medically attended outpatient RSV cases per season, corresponding to an estimated societal burden of €190.9 million per RSV season.

C. Additional scenario analysis excluding indirect costs

Rationale

In the base-case analysis, costs were estimated from a societal perspective and therefore included direct reimbursed medical costs, patient-borne out-of-pocket costs, and indirect costs. Indirect costs were mainly driven by productivity losses and occurred only in the subgroup of patients who were still in paid employment. Because the RSV-positive outpatient cohort consisted of adults aged ≥60 years, productivity-loss costs were expected to be concentrated in a minority of economically active patients. An additional scenario analysis was therefore performed to assess the influence of indirect costs on the national burden estimates.

Analytical approach

The scenario analysis used the same two national case-number extrapolations as the base-case analysis:

1. Incidence-based extrapolation: 258,206 medically attended outpatient RSV cases per season.
2. ARE–prevalence-based extrapolation: 374,395 medically attended outpatient RSV cases per season.

In the base-case analysis, the mean societal cost per outpatient RSV episode was €510.0 and included direct reimbursed medical costs of €161.3 per episode, indirect costs of €331.8 per episode, and patient-borne out-of-pocket costs of €16.9 per episode.

For the scenario analysis, indirect costs were excluded. The cost per episode was therefore recalculated as:

Cost per episode excluding indirect costs = direct reimbursed medical costs + out-of-pocket costs

Cost per episode excluding indirect costs = €161.3 + €16.9

Cost per episode excluding indirect costs = €178.2

Thus, the scenario analysis reflects the estimated outpatient cost burden from a direct medical and patient-borne cost perspective, excluding productivity losses and other indirect costs.

Incidence-based extrapolation

Using the incidence-based estimate of 258,206 medically attended outpatient RSV cases per season, the cost burden excluding indirect costs was calculated as:

258,206 × €178.2 = €46,012,309

This corresponds to approximately €46.0 million per RSV season.

For comparison, the corresponding base-case societal burden estimate was:

258,206 × €510.0 = €131,685,060

This corresponds to approximately €131.7 million per RSV season.

ARE–prevalence-based extrapolation

Using the ARE–prevalence-based estimate of 374,395 medically attended outpatient RSV cases per season, the cost burden excluding indirect costs was calculated as:

374,395 × €178.2 = €66,717,189

This corresponds to approximately €66.7 million per RSV season.

For comparison, the corresponding base-case societal burden estimate was:

374,395 × €510.0 = €190,941,450

This corresponds to approximately €190.9 million per RSV season.

Summary of the scenario analysis

The base-case societal analysis yielded an estimated national outpatient RSV burden of €131.7 million to €190.9 million per season. When indirect costs were excluded, the estimated burden decreased to €46.0 million to €66.7 million per season.

**Supplementary References**

1. Kassenärztliche Bundesvereinigung. Einheitlicher Bewertungsmaßstab (EBM) [Uniform Value Scale (EBM)] [Internet]. Berlin: Kassenärztliche Bundesvereinigung; [cited 2026 Mar 31]. Available from: [https://www.kbv.de/praxis/abrechnung/ebm](https://www.kbv.de/praxis/abrechnung/ebm?utm_source=chatgpt.com)
2. Bundesministerium für Gesundheit. Zuzahlung und Erstattung von Arzneimitteln [Copayments and reimbursement of medicinal products] [Internet]. Berlin: Bundesministerium für Gesundheit; [cited 2026 Mar 31]. Available from: [https://www.bundesgesundheitsministerium.de/zuzahlung-und-erstattung-arzneimittel](https://www.bundesgesundheitsministerium.de/zuzahlung-und-erstattung-arzneimittel?utm_source=chatgpt.com)
3. Bundesministerium der Finanzen. Lohnsteuer-Hinweise 2025: Pauschale Kilometersätze [Wage Tax Guidelines 2025: Flat-rate mileage allowances] [Internet]. Berlin: Bundesministerium der Finanzen; 2025 [cited 2026 Mar 31]. Available from: [https://lsth.bundesfinanzministerium.de/lsth/2025/A-Einkommensteuergesetz/II-Einkommen-2-24b/4-Ueberschuss-d-Einnahmen-ueber-die-Werbungsk-8-9a/Paragraf-9/h-9-5.html](https://lsth.bundesfinanzministerium.de/lsth/2025/A-Einkommensteuergesetz/II-Einkommen-2-24b/4-Ueberschuss-d-Einnahmen-ueber-die-Werbungsk-8-9a/Paragraf-9/h-9-5.html?utm_source=chatgpt.com)
4. Koopmanschap MA, van Exel JN, van den Berg B, Brouwer WB. An overview of methods and applications to value informal care in economic evaluations of healthcare. Pharmacoeconomics. 2008;26(4):269-80. doi:10.2165/00019053-200826040-00001.
5. van den Berg B, Brouwer W, van Exel J, Koopmanschap M, van den Bos GA, Rutten F. Economic valuation of informal care: lessons from the application of the opportunity costs and proxy good methods. Soc Sci Med. 2006;62(4):835-45. doi:10.1016/j.socscimed.2005.06.046.
6. Statistisches Bundesamt (Destatis). Inlandsproduktsberechnung – Detaillierte Jahresergebnisse (endgültige Ergebnisse), Fachserie 18 Reihe 1.4 [National accounts – Detailed annual results (final results), Series 18, Row 1.4] [Internet]. Wiesbaden: Statistisches Bundesamt; 2025. Available from: [https://www.destatis.de/DE/Themen/Wirtschaft/Volkswirtschaftliche-Gesamtrechnungen-Inlandsprodukt/Publikationen/Downloads-Inlandsprodukt/inlandsprodukt-endgueltig-xlsx-2180140.html](https://www.destatis.de/DE/Themen/Wirtschaft/Volkswirtschaftliche-Gesamtrechnungen-Inlandsprodukt/Publikationen/Downloads-Inlandsprodukt/inlandsprodukt-endgueltig-xlsx-2180140.html?utm_source=chatgpt.com)
7. Graf von der Schulenburg JM, Greiner W, Jost F, et al.; Hanover Consensus Group. German recommendations on health economic evaluation: third and updated version of the Hanover Consensus. Value Health. 2008;11(4):539-44. doi:10.1111/j.1524-4733.2007.00301.x.
8. Statistikportal. Arbeitnehmerentgelt, Bruttolöhne und -gehälter in den Ländern der Bundesrepublik Deutschland 1991 bis 2024 [Compensation of employees and gross wages and salaries in the federal states of Germany, 1991 to 2024] [Internet]. Düsseldorf: Statistikportal; 2025 [cited 2026 Mar 31]. Available from: [https://www.statistikportal.de/de/veroeffentlichungen/arbeitnehmerentgelt-bruttoloehne-und-gehaelter](https://www.statistikportal.de/de/veroeffentlichungen/arbeitnehmerentgelt-bruttoloehne-und-gehaelter?utm_source=chatgpt.com)
9. Minijob-Zentrale. Mindestlohn im Minijob [Minimum wage in mini-jobs] [Internet]. Essen: Minijob-Zentrale; [cited 2026 Mar 31]. Available from: [https://www.minijob-zentrale.de/DE/die-minijobs/arbeitsrechte-im-minijob/mindestlohn](https://www.minijob-zentrale.de/DE/die-minijobs/arbeitsrechte-im-minijob/mindestlohn?utm_source=chatgpt.com)
10. Goerlitz L, Tolksdorf K, Prahm K, et al. ARE-Konsultationsinzidenz [Acute respiratory illness consultation incidence] [dataset]. Zenodo; 2026. Available from: https://doi.org/10.5281/zenodo.19231008
11. Statistisches Bundesamt (Destatis). GENESIS-Online Datenbank, Tabelle 12411-0005: Bevölkerung: Deutschland, Stichtag, Altersjahre [GENESIS-Online database, table 12411-0005: Population of Germany by reference date and age in years] [Internet]. Wiesbaden: Statistisches Bundesamt; [cited 2026 Mar 31]. Available from: [https://www-genesis.destatis.de/genesis/online](https://www-genesis.destatis.de/genesis/online?utm_source=chatgpt.com)
12. Statistisches Bundesamt (Destatis). Bevölkerungsstand: Amtliche Einwohnerzahl Deutschlands [Population: official population count of Germany] [Internet]. Wiesbaden: Statistisches Bundesamt; [cited 2026 Mar 31]. Available from: [https://www.destatis.de/DE/Themen/Gesellschaft-Umwelt/Bevoelkerung/Bevoelkerungsstand/_inhalt.html](https://www.destatis.de/DE/Themen/Gesellschaft-Umwelt/Bevoelkerung/Bevoelkerungsstand/_inhalt.html?utm_source=chatgpt.com)
13. BUCOSS Final Interim Report. Observation period covering the three infection seasons 2022/23, 2023/24, and 2024/25, each running from October to April.
