# Supplementary material for: The outpatient economic burden of respiratory syncytial virus infection in adults aged ≥ 60 years in Germany: results from the BUCOSS study
Source: Respir Res. 2026 Aug 14;27:344. doi: 10.1186/s12931-026-03866-1 (PMC13546509; doi:10.1186/s12931-026-03866-1)
Supplement: Supplementary file 2 [file 12931_2026_3866_MOESM2_ESM.docx]

**Supplementary Appendix S2**

**English translation of the cost data collection forms used in the BUCOSS study**

The following English translation of the cost data collection forms used in the BUCOSS study is provided to improve the transparency and reproducibility of the health-economic data collection. The translated forms correspond exclusively to the cost-related sections of the original German study documentation. The wording, sequence of questions, response options and input fields were retained as closely as possible to the original forms. Data collection during the study was performed exclusively using the German-language version. Repeated questions at follow-up visits are intentionally reproduced because the same resource utilisation variables were prospectively assessed at each visit.

**Abbreviations used in this appendix:** GP, general practitioner; KV emergency practice, outpatient emergency practice operated within the German statutory health insurance system; OTC, over-the-counter; RSV, respiratory syncytial virus.

## A. Baseline visit (Day 0)

### A1. Employment and productivity loss

**1. Are you currently working (paid work)?**

☐ Yes	☐ No

**2. Which of the following statements about your employment is applicable?**

☐ 450-euro job	☐ Mini-job	☐ Full-time employment	☐ Part-time employment

If mini-job: monthly income € ______

If part-time employment: employment level ______ %

**3. How many working hours have you missed during the last ten days (including today) because of problems related to your respiratory illness?**

Number of working hours missed: ______ hours

### A2. Travel to the participating practice at baseline

*The following items refer to the baseline consultation at the participating practice.*

**1. Did you travel to the practice by private car?**

☐ Yes	☐ No

If yes: one-way distance from your home to the practice: ______ km

**2. Did you travel to the practice by taxi?**

☐ Yes	☐ No

If yes: costs incurred (rounded): € ______

**3. Were you driven to the practice by acquaintances or relatives (excluding taxi)?**

☐ Yes	☐ No

If yes: duration of the journey: ______ minutes

**4. Did you incur parking fees because of the trip to the practice?**

☐ Yes	☐ No

If yes: costs incurred: € ______

**5. Did you have to make statutory co-payments for prescribed medications?**

☐ Yes	☐ No

If yes: number of medications requiring a co-payment: ______

Total co-payment amount: € ______

### A3. Previous physician consultations during the ten days before baseline

**1. During the last ten days, have you already sought medical help in the current practice or another practice because of your respiratory infection?**

☐ Yes	☐ No

**Which physician or facility did you consult?**

☐ GP (general practitioner; primary-care internist)	☐ Pulmonologist	☐ Physicians at a hospital outpatient department / KV emergency practice	☐ Other

If other: please specify: ______________________________

*If medical help had already been sought during the previous ten days, the following cost items were recorded for the corresponding consultation:*

**1. Did you travel to the practice by private car?**

☐ Yes	☐ No

If yes: one-way distance from your home to the practice: ______ km

**2. Did you travel to the practice by taxi?**

☐ Yes	☐ No

If yes: costs incurred (rounded): € ______

**3. Were you driven to the practice by acquaintances or relatives (excluding taxi)?**

☐ Yes	☐ No

If yes: duration of the journey: ______ minutes

**4. Did you incur parking fees because of the trip to the practice?**

☐ Yes	☐ No

If yes: costs incurred (rounded): € ______

**5. Did you have to make statutory co-payments for prescribed medications?**

☐ Yes	☐ No

If yes: number of medications requiring a co-payment: ______

Total co-payment amount: € ______

### A4. Over-the-counter medication before the index consultation

**Have you already taken non-prescription medication because of your respiratory infection during the last ten days before the physician visit?**

☐ Yes	☐ No

Medication(s), if applicable: __________________________________________

Medication group, if applicable: ______________________________________

## B. Follow-up visit (Day 14)

### B1. Employment and productivity loss

**1. Are you currently working (paid work)?**

☐ Yes	☐ No

**3. How many working hours have you missed during the last ten days (including today) because of problems related to your respiratory illness?**

Number of working hours missed: ______ hours

### B2. Additional medical care since study enrolment

**1. During the last 14 days (since the start of the study), have you again sought medical help because of your respiratory infection, for example at your GP practice, specialist practice or an emergency department?**

☐ Yes	☐ No

**2. How often?**

Number of consultations: ______

*For each additional physician consultation reported at Day 14 (physician visit x), the following items were recorded:*

**1. Did you travel to the practice by private car?**

☐ Yes	☐ No

One-way distance from your home to the practice: ______ km

**2. Did you travel to the practice by taxi?**

☐ Yes	☐ No

Costs incurred (rounded): € ______

**3. Were you driven to the practice by acquaintances or relatives (excluding taxi)?**

☐ Yes	☐ No

Duration of the journey: ______ minutes

**4. Did you incur parking fees because of the trip to the practice?**

☐ Yes	☐ No

Costs incurred: € ______

**5. Did you have to make statutory co-payments for prescribed medications?**

☐ Yes	☐ No

Number of medications requiring a co-payment: ______

Total co-payment amount: € ______

## C. Follow-up visit (Day 28)

### C1. Employment and productivity loss

**1. Are you currently working (paid work)?**

☐ Yes	☐ No

**3. How many working hours have you missed during the last ten days (including today) because of problems related to your respiratory illness?**

Number of working hours missed: ______ hours

### C2. Additional medical care during the follow-up period

**1. During the last 14 days (since the start of the study), have you again sought medical help because of your respiratory infection, for example at your GP practice, specialist practice or an emergency department?**

☐ Yes	☐ No

**2. How often?**

Number of consultations: ______

*For each additional physician consultation reported at Day 28 (physician visit x), the following items were recorded:*

**1. Did you travel to the practice by private car?**

☐ Yes	☐ No

One-way distance from your home to the practice: ______ km

**2. Did you travel to the practice by taxi?**

☐ Yes	☐ No

Costs incurred (rounded): € ______

**3. Were you driven to the practice by acquaintances or relatives (excluding taxi)?**

☐ Yes	☐ No

Duration of the journey: ______ minutes

**4. Did you incur parking fees because of the trip to the practice?**

☐ Yes	☐ No

Costs incurred: € ______

**5. Did you have to make statutory co-payments for prescribed medications?**

☐ Yes	☐ No

Number of medications requiring a co-payment: ______

Total co-payment amount: € ______

## D. Physician-prescribed medication and other therapies

### D1. Physician-prescribed medication

*For each prescribed medicinal product x, the following information was documented:*

Prescribed medicinal product x (selection from Rote Liste): __________________________________

Prescribed medicinal product x (Rote Liste code): __________________________________________

**Route of administration for medicinal product x**

☐ Oral	☐ Intravenous	☐ Inhaled	☐ Subcutaneous	☐ Rectal	☐ Sublingual	☐ Intranasal	☐ Other

**Package size for medicinal product x**

☐ N1	☐ N2	☐ N3

Treatment start date: ____ / ____ / ______

**Ongoing treatment?**

☐ Yes	☐ No

Treatment end date if treatment is not ongoing: ____ / ____ / ______

### D2. Other therapies

**Other forms of therapy?**

☐ Yes	☐ No

If yes: other therapy form 1: __________________________________________

## Note on repeated entries

*Variables marked with x in the source documentation indicate fields that could be repeated when more than one physician consultation, medication or therapy was documented for the same patient. The repeated structure has been retained in this English translation.*
